# IL-36-related genes predict prognosis of gastric cancer

**DOI:** 10.3389/fonc.2025.1566993

**Published:** 2025-06-18

**Authors:** Ying Zhang, Yanbo Liu, Xin Guan, Meng Qu, Dianxiu Wu, Ning Liu, Zhengkun Lin, Yuqi Liu, Han Wang, Lijuan Yang

**Affiliations:** ^1^ Department of Pathology, Affiliated Hospital, Beihua University, Jilin, China; ^2^ Department of Pathophysiology, Basic medical college, Beihua University, Jilin, China; ^3^ Department of Cell Biology, Basic medical college, Beihua University, Jilin, China; ^4^ Department of Biochemistry and Molecular Biology, Basic medical college, Beihua University, Jilin, China; ^5^ Department of Physiology, Basic medical college, Beihua University, Jilin, China; ^6^ Department of Biochemistry Laboratory, Basic medical college, Beihua University, Jilin, China

**Keywords:** GC, IL-36RDEGs, prognosis model, risk score, prediction

## Abstract

**Introduction:**

Gastric cancer (GC) is one of the most frequently encountered malignant tumors in the clinic. Because effective early screening techniques are lacking, most patients have advanced disease at first diagnosis. The interleukin (IL)-36 family plays a vital role in regulating the immune system, inflammatory responses, and the occurrence and development of cancer. Hence, this study explored the potential role of IL-36 related genes (IL-36RGs) in GC and built a prognostic risk assessment model for GC based on IL-36RGs, which can help evaluate treatment and prognosis.

**Methods:**

First, relevant datasets were downloaded from public databases. After processing the datasets to remove batch effects, perform differential analysis, and take intersections, IL-36-related differentially expressed genes (IL-36RDEGs) were screened. A prognostic risk model containing nine model genes was constructed based on univariate Cox and least absolute shrinkage and selection operator (LASSO) regression methods. Then, to investigate the potential biological activities of the model genes in GC, we conducted enrichment, PPI interaction network, and immune infiltration analyses. Immunohistochemical staining was conducted to validate the expression of IL-36A in GC.

**Results:**

The prognostic risk model analysis revealed that mortality events in the high-risk group were substantially elevated compared to those in the low-risk group. The model demonstrated excellent predictive capability at 1, 2, and 3 years and showed the best clinical predictive performance at 3 years. Bioinformatics analysis of the model genes indicate that they primarily participate in mechanisms that promote the synthesis and secretion of cytokines in GC. And hub genes may be strongly correlated with host immune response mechanisms. According to the immunohistochemical staining results, IL-36A expression was higher in the STAD group than in the control group.

**Conclusions:**

The results of the above analysis suggest that IL-36RDEGs can serve as independent prognostic biomarkers for GC and provide insights into IL-36RGs from both bioinformatics and experimental validation perspectives.

## 1 Introduction

Gastric cancer (GC) is one of the most prevalent malignant tumors encountered in clinical practice. Statistical data show that GC ranks fifth as the most frequently occurring cancer and fourth leading cause of death, especially in East Asia and Eastern Europe, where it has the highest incidence and usually a poor prognosis (1). Owing to the lack of obvious symptoms in early-stage patients and the insidious course of the disease, including the current lack of effective early screening methods in clinical practice, the majority of patients are found to be in an advanced stage upon their initial diagnosis (2). Although the pathogenesis of GC is not completely understood, its growth and differentiation are regulated by various factors. Most clinical treatments include surgical resection, targeted therapy, systemic chemotherapy, and radiotherapy. Although these treatment methods are effective (3, 4), the long-term survival outcomes for patients with GC are unsatisfactory, especially for patients with advanced GC (5). Therefore, identification of new prognostic biomarkers and molecular targets is urgently required to better predict the prognosis of patients with GC and guide individualized clinical diagnosis and treatment.

IL-36 belongs to the IL-1 family of cytokines, including three isoforms: IL-36A (IL-36α), IL-36B (IL-36β) and, IL-36G (IL-36γ). It forms a complex by binding to IL-36R (IL-1RL2) and recruiting IL-1 receptor accessory protein (IL-1RAcP), it subsequently triggers the activation of associated signaling pathways, including MyD88-NF-κB (Myeloid differentiation primary response 88) and Mitogen-activated protein kinase (MAPK), which modulate the expression of downstream target genes (6); IL-36Ra and IL-38 are antagonists of IL-36R; upon binding to IL-36R, they prevent the recruitment of IL-1RAcP, thereby inhibiting the assembly of an active IL-36R complex and limiting signal transduction (7, 8). Studies have shown that IL-36 and its related signaling pathways are associated with various inflammatory diseases, autoimmune diseases, and cancers (9–12). The study found that, compared to adjacent tissues, the expression of IL-36 is significantly reduced in human hepatocellular carcinoma (HCC) tissues. HCC cases with IL-36 positive expression have a lower recurrence rate and longer survival time (13). IL-36A inhibits HCC proliferation, survival, and migration, which correlates with a decrease in the expression of cytokines IL-1β and IL-18, suggesting that IL-36A may inhibit pyroptosis (14). In contrast to non-cancerous tissues, the mRNA and protein expression of IL-36 family members is increased in colorectal cancer tissues, and IL-36R can activate the oncogenic phenotype of cancer cells (11, 15). The relationship between IL-36RGs and tumors, whether inhibitory or promotional, may be related to different types of cancer, various samples, and the heterogeneity of the cancer itself. Over the last few years, studies have revealed a significant association between chronic inflammation in the tumor microenvironment and cancer, with IL-36 considered to play a major role in aseptic chronic inflammation (8, 16). A studies has shown that IL-36 is a potent activator of innate immune cells and mediates a strong anti-tumor response through complement and adaptive immunity. IL-36-treated neutrophils can directly kill tumor cells, induce NK cells to generate cytolytic activity, and enhance T cell proliferation. The interactions between these IL-36-treated neutrophils and other immune components in the tumor microenvironment (TME) result in a highly effective anti-tumor response (17). Research has confirmed that IL-36β can promote the activation of CD8+ T cells by activating mTORC1 through PI3K/Akt, IKK, and MyD88 pathways, thereby enhancing the anti-tumor immune response and laying the groundwork for the application of IL-36β in tumor immunotherapy (18). However, the relationship between IL-36RGs and GC has rarely been studied, and their mechanism of action remains unclear.

To reveal the relationship between IL-36RGs and GC, this study integrated and analyzed data from multiple public databases, including TCGA-STAD, GSE19826, and GSE54129. By implementing strict data preprocessing techniques (such as batch effect correction and differential analysis), we obtained IL-36RDEGs. Based on this, we constructed a prognostic risk model using various statistical methods, including univariate Cox regression and LASSO regression, and performed external validation with an independent GEO dataset to ensure the robustness and wide applicability of the study results. Through systematic multi-cohort cross-validation, this research can contribute to a comprehensive assessment of the role of IL-36RDEGs in GC prognosis and their potential mechanisms, thereby providing a theoretical basis for the clinical development of new prognostic biomarkers and molecular therapeutic targets.

## 2 Materials and methods

### 2.1 Technical roadmap

**Figure 1:**
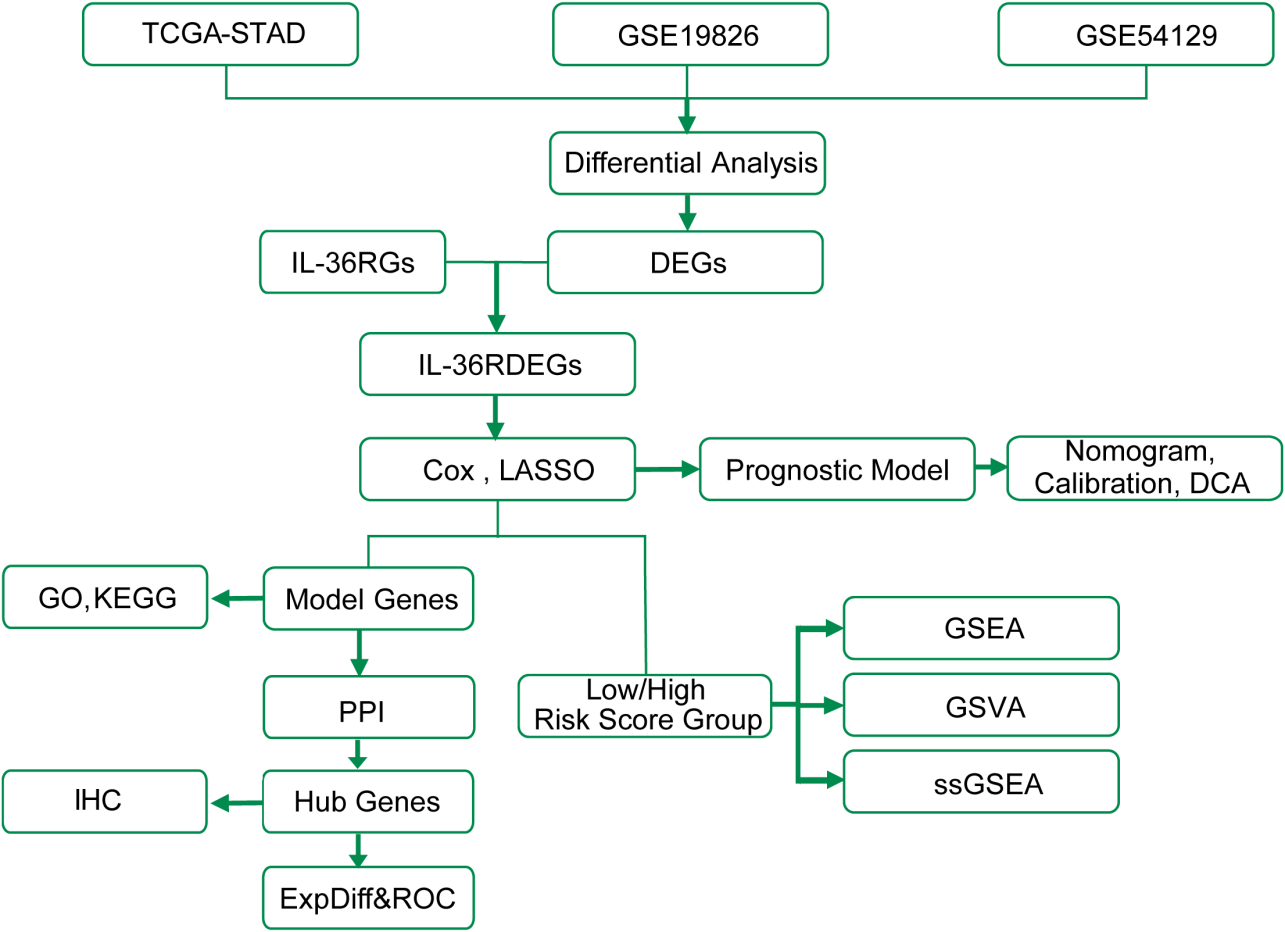
Work flow diagram of this study. TCGA, The Cancer Genome Atlas; STAD, Stomach adenocarcinoma; DEGs, Differentially Expressed Genes; GSEA, Gene Set Enrichment Analysis; GO, Gene Ontology; KEGG, Kyoto Encyclopedia of Genes and Genomes; ExpDiff&ROC, Expression Differential & Receiver Operating Characteristic; LASSO, Least Absolute Shrinkage and Selection Operator; DCA, Decision Curve Analysis; GSVA, Gene Set Variation Analysis; ssGSEA, single-sample Gene-Set Enrichment Analysis; PPI, Protein-Protein Interaction; IHC, Immunohistochemistry.

The technical roadmap is shown (**Figure 1**).

### 2.2 Data download

The TCGA-STAD dataset was sourced from the TCGA database (https://portal.gdc.cancer.gov/) with the aid of the R package TCGAbiolinks (19) and was used as the test set, excluding samples that did not have clinical details. After normalization, the associated clinical data were extracted from the UCSC Xena database (20) (https://xena.ucsc.edu/) and are summarized in **Table 1**. The GSE19826 and GSE54129 datasets were obtained from the GEO database (21) (https://www.ncbi.nlm.nih.gov/geo/) using the GEOquery (22) package in R for further validation purposes (23). The sva package in R was used to eliminate batch effects from the data (24). The GSE19826 and GSE54129 datasets were processed for probe annotation, standardization, normalization, and other treatments using the R package limma (25). All selected STAD and control samples were used in this study. Further information is provided in **Table 2**. A total of 44 IL-36RGs were sourced from the GeneCards (26) (https://www.genecards.org/) and MsigDB (27) (Molecular Signatures Database). After using the term “Interleukin-36” as a search keyword and keeping only “protein-coding” IL-36RGs, a total of 44 IL-36RGs were obtained in the GeneCards. In addition, we also got seven IL-36RGs in MsigDB database. IL-36RGs obtained in the above way were merged and deduplicated to obtain a total of 44 IL-36RGs.

**Table 1:** Overall baseline data sheet.

Characteristics	Overall
Age, n (%)	
>60	222 (66.3%)
<=60	113 (33.7%)
Gender, n (%)	
MALE	214 (63.9%)
FEMALE	121 (36.1%)
Pathologic_stage, n (%)	
Stage III	146 (43.6%)
Stage II	108 (32.2%)
Stage IV	34 (10.1%)
Stage I	47 (14%)

**Table 2:** GEO microarray chip information.

Information about the dataset	GSE19826	GSE54129
Platform	GPL570	GPL570
Species	Homo sapiens	Homo sapiens
Tissue	Gastric Tissue	Gastric Tissue
Samples in GC group	12	111
Samples in Control group	15	21
Reference	21132402	–

### 2.3 IL-36-related differentially expressed genes

We employed the limma package in R to perform differential analysis of genes in the STAD and Control groups across the TCGA-STAD, GSE19826, and GSE54129 datasets. We set the thresholds for DEGs as |log2FC| > 0 and *p* value < 0.05, and the outcomes of the differential analysis were plotted as a volcano plot using the ggplot2 package in R. We intersected the DEGs obtained from the differential analysis in the TCGA-STAD dataset with IL-36RGs and obtain IL-36RDEGs to draw a Venn diagram, which was then displayed as a heatmap using the pheatmap package in R.

### 2.4 Creation of a risk model for predicting prognosis related to GC

Using the R package survival (28), the prognostic risk model in the TCGA-STAD dataset was created to analyze the effect of IL-36RDEGs on prognosis through single and multivariate Cox regression analyses based on clinical information and to ascertain whether IL-36RDEGs are an independent prognostic factor. The process began with univariate Cox regression analysis of genes showing *p* value < 1, after which LASSO regression analysis with family = “cox” applying the package glmnet in R (29) was conducted to ascertain the model genes for the prognostic risk model. We adopted a 10-fold cross-validation approach to determine the variables in the LASSO regression model and identify the optimal penalty parameter λ. After completing the training for all 10 folds, we calculated the average of C-index to ascertain the optimal λ that produced the highest mean C-index. This optimal λ was employed to construct the LASSO regression model, facilitating the selection of the most significant genes.

Finally, using the LASSO risk score and clinical information, we conducted a multivariate Cox regression analysis. The following approach was used to compute risk scores:


$$\text{riskScore}\;=\;\sum_{\text{i}}\text{Coefficient}\;\left({\text{gene}}_{\text{i}}\right)*\text{mRNA}\;\text{Expression}\;\left({\text{gene}}_{\text{i}}\right)$$

Furthermore, a risk factor graph was created using the LASSO risk score and the package ggplot2 in R.

This study sought to explore the differences in overall survival (OS) between high-risk (High) and low-risk (Low) patients in the STAD group of the E dataset. Using the R package survival, we performed a Kaplan–Meier (KM) (30) curve analysis and plotted the KM curves according to the LASSO risk score. Then, the survivalROC package in R (31) was employed to generate time-dependent ROC curves utilizing the LASSO risk score and OS data, and the area under the curve (AUC) was computed to estimate the survival outcomes for 1, 2, and 3-year periods within the STAD group from TCGA-STAD dataset.

### 2.5 Analysis of enrichment in GO and KEGG pathways

To further investigate the biological and signaling pathways associated with the genes linked to the risk score, we performed enrichment analysis using Gene Ontology (GO) (32) and the Kyoto Encyclopedia of Genes and Genomes (KEGG) (33). The model genes were subjected to GO and KEGG enrichment analysis using the clusterProfiler (34) package in R, utilizing entry screening criteria that considered a *p* value < 0.05 and an FDR value (*q* value) < 0.25 as statistically significant. Finally, the GO and KEGG enrichment analysis results were visualized using the R package PathView (35).

### 2.6 Gene set enrichment analysis

In the TCGA-STAD dataset, the STAD group was sorted into high- and low-risk groups using the median value of the LASSO risk score, and GSEA was performed for all genes in the STAD group using the clusterProfiler package in R (34). The GSEA screening criteria were set as adj.*p* < 0.05 and an FDR value (*q* value) < 0.25, correcting the *p* value using the Benjamini–Hochberg method.

### 2.7 Gene set variant analysis

The h.all.v7.4.symbols.gmt gene set was sourced from the MSigDB database, and GSVA (36) was conducted on all genes in the TCGA-STAD dataset to evaluate the differences in enrichment of functions among the high- and low-risk groups, using a GSVA selection criterion of *p* value < 0.05. This was performed to assess whether the various samples were enriched in different pathways.

### 2.8 Assessment of the prognostic risk model for GC

To illustrate the findings of the multivariate Cox regression analysis, a forest plot was created to represent the expression of the LASSO risk score and clinical variables considered in the analysis. A nomogram (37) was created based on the outcomes of the multivariate Cox regression analysis using the rms package in R, illustrating the correlation between clinical information and the LASSO risk score inside the multivariate Cox regression model. Calibration analysis was conducted to draw calibration curves and evaluate the accuracy and discrimination capacity of the prognostic risk model based on the LASSO risk score. Applying the R package ggDCA, a decision curve analysis (DCA) (38) diagram derived from the LASSO risk score was generated to assess the accuracy and discrimination of the prognostic risk model for GC.

### 2.9 PPI interaction network and functional similarity analysis

Using the STRING database (39), hub genes were screened, a hub gene-related protein-protein interaction network (PPI Network) was created, and the PPI network model was visualized using Cytoscape (40). Using the GeneMANIA database (41) (http://genemania.org), we predicted genes with functions analogous to the hub genes online and downloaded the interaction network. The inner circle in the figure represents the hub genes in our study, and the outer circle represents functionally similar genes.

### 2.10 Validation of differential expression of hub genes and ROC curve analysis

To further explore the differences in hub gene expression in the TCGA-STAD dataset and in STAD and Controls in the GSE19826 and GSE54129 datasets, group comparison plots were constructed based on the expression of hub genes. Finally, the package pROC in R was employed to generate the ROC curve for the hub genes and to compute the AUC to assess the diagnostic impact of hub gene expression levels on the occurrence of GC. Subsequently, to investigate the connections among hub genes, the Spearman algorithm was employed to assess the association between hub gene expression and TCGA-STAD dataset. A heat map generated using the pheatmap package in R represents the outcomes of the relationship analysis.

### 2.11 Immune infiltration examination of the high- and low-risk groups

First, various infiltrating immune cell subtypes were identified. Subsequently, the enrichment scores derived from ssGSEA (42) represented the comparative immune cell infiltration abundance in each sample, which produced an immune cell infiltration matrix for the STAD group in the TCGA-STAD dataset. The package ggplot2 in R was used to demonstrate the differences in immune cell expression between the high- and low-risk groups within the STAD group of TCGA-STAD dataset. The immune cells showing considerable differences between the two groups were filtered for additional analysis; the correlation between immune cells and hub genes was assessed using the Spearman algorithm; and the findings were depicted through a correlation bubble map produced with the R package ggplot2.

### 2.12 Patient and tissue samples

This study was approved by the Ethics Committee of the Affiliated Hospital of Beihua University in Jilin City, Jilin Province, China (Approval No. 20240084). All patients signed an informed consent form. This study used GC and adjacent tissue samples collected by our research group with clear pathological diagnoses. All patients with GC underwent radical surgery and did not receive endocrine or radiation therapy prior to surgery.

### 2.13 Immunohistochemical staining and evaluation methods

Immunohistochemistry was conducted using an IL-36A antibody (24173-1-AP; Proteintech, China) diluted at 1:100, following the manufacturer’s instructions and based on preliminary experiments. Two pathologists independently assessed the pathological grouping of each slice. The positive expression rate of the tissue slices was calculated using the ImageJ software. The IL-36A positive expression rate was calculated as follows: Positive area/Total area × 100%.

### 2.14 Statistical analysis

The R software was used to analyze all data in this study (Version 4.4.1). Unless otherwise specified, statistical significance for comparisons of two categories of continuous variables was examined by applying the independent Student’s t-test for normally distributed variables and the Mann–Whitney U test, commonly known as the Wilcoxon rank-sum test, for variables that were not normally distributed. The Kruskal–Wallis test was used to assess differences across three or more groups. Correlation coefficients between different molecules were calculated using Spearman’s correlation analysis. All statistical *p*-values were calculated using a two-tailed method, with a *p* value < 0.05, considered statistically meaningful. Quantitative data are represented as mean ± standard deviation (SD).

## 3 Results

### 3.1 Processing of GC datasets

**Figure 2:**
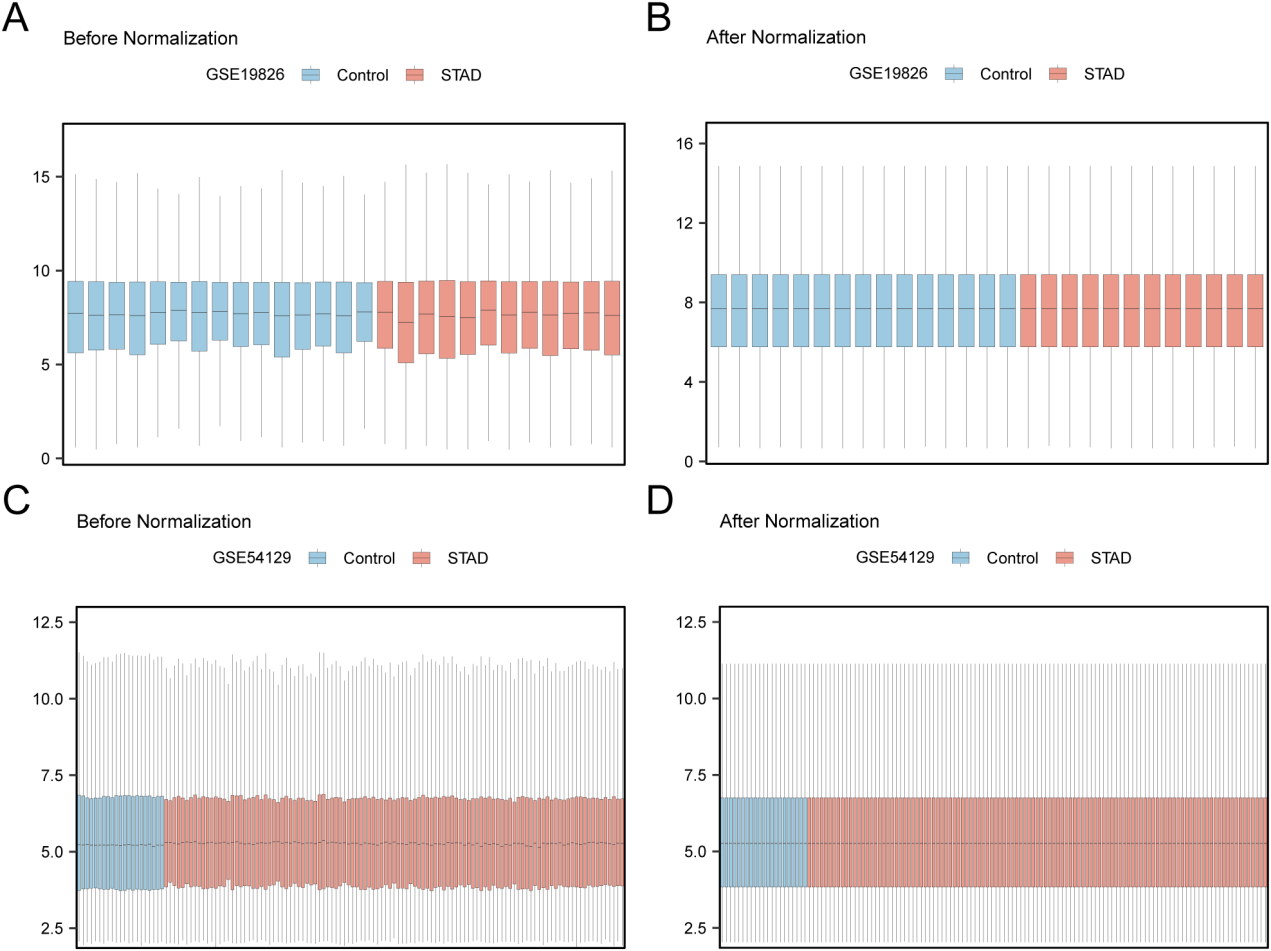
Batch Effects Removal of GSE19826 and GSE54129. **(A)** Distribution boxplot of GSE19826 dataset before going batch. **(B)** Distribution boxplot of the post-batch GSE19826 dataset. **(C)** The distribution of GSE54129 dataset boxplot before batch processing. **(D)** The distribution boxplot of the post-batch GSE54129 dataset.

First, batch effects in the GSE19826 and GSE54129 datasets were eliminated using the R package sva. Subsequently, boxplots were generated to compare the differences in illustration values of the datasets before and after batch-effect removal (**Figures 2A–D**). The outcomes from the box plots indicate that the batch effects among the samples in the datasets were largely eradicated after batch removal.

### 3.2 Differentially expressed genes related to IL-36

**Figure 3:**
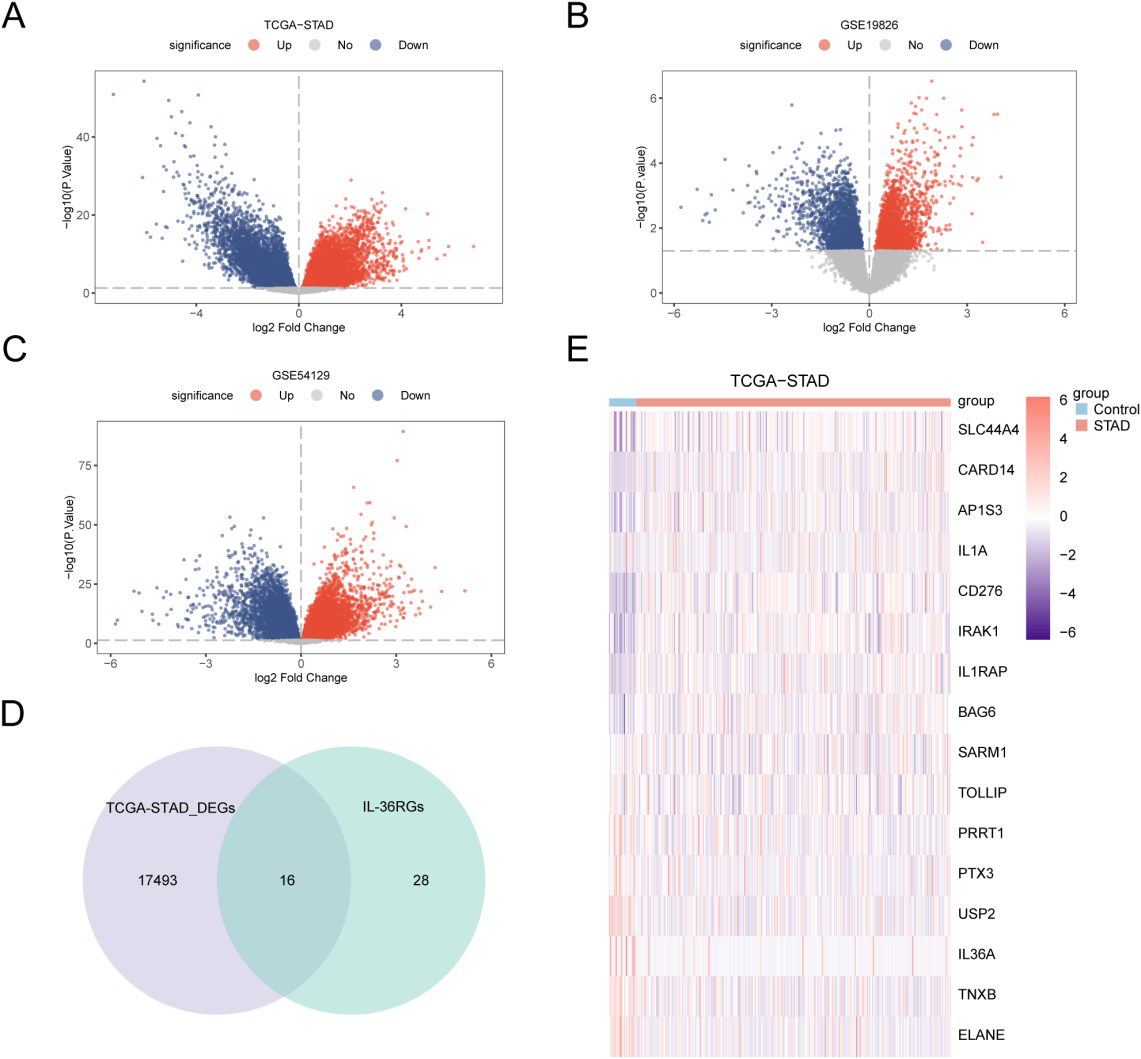
Differential Gene Expression Analysis. **(A-C)** Volcano plot of DEGs analysis between STAD and Control groups in the TCGA-STAD, GSE19826 and GSE54129 datasets. **(D)** The DEGs in the TCGA-STAD dataset and IL-36RGs Wayne figure. **(E)** Heatmap of the expression of IL-36RDEGs in the TCGA-STAD dataset.

Differential analysis was conducted on STAD and control samples from the TCGA-STAD dataset, together with the GSE19826 and GSE54129 datasets, applying the R package limma to identify DEGs, and volcano plots were created from the results of each dataset’s differential analysis (**Figures 3A–C**). The intersections of all DEGs from the TCGA-STAD dataset, which fulfilled the thresholds of |log2FC| > 0 and p < 0.05, and IL-36RGs were selected. A Venn diagram was plotted showing 16 IL-36RDEGs (**Figure 3D**). Following intersection analysis, the differences of IL-36RDEGs in various sample groups within the TCGA-STAD dataset were evaluated, and a heatmap was generated using the package pheatmap in R to illustrate the results (**Figure 3E**).

### 3.3 Construction of a prognostic risk model for GC

**Figure 4:**
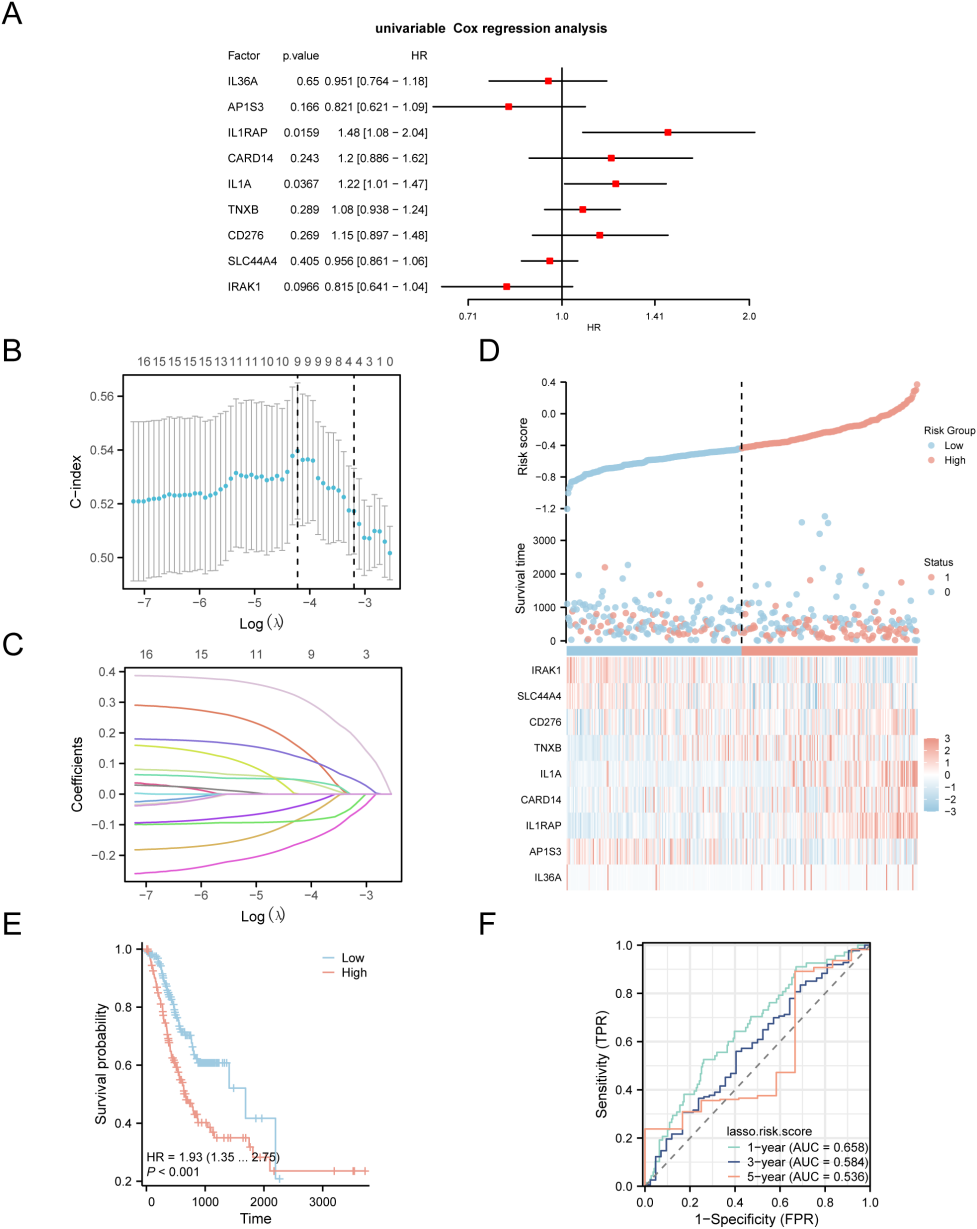
LASSO and Cox Regression Analysis. **(A)** Forest plot of the univariate Cox regression analysis of the prognosis of IL-36RDEGs. **(B, C)** Prognostic risk model plot and variable trajectory plot of the LASSO regression model. **(D)** Risk factor plot of Model Genes prognostic LASSO model. **(E)** Prognostic KM curves between high-risk and low-risk groups of LASSO risk score and OS in the STAD group. **(F)** Time-dependent ROC curve of STAD group in the TCGA-STAD dataset. *p* value < 0.001, extremely statistically significant.


To develop a prognostic risk model for GC, we conducted univariate Cox regression analysis using clinical information from the STAD group in the TCGA-STAD dataset combined with IL-36RDEGs. The LASSO regression analysis included all variables with a *p* value < 1 from the univariate analysis, and a forest plot was constructed to present the outcomes (**Figure 4A**). To further determine the prognostic value of the genes from the univariate Cox regression model in GC, LASSO regression analysis was conducted and a LASSO regression model was constructed. The C-index was used as the criterion for selecting LASSO variables, and the results were visually presented in the LASSO regression model (**Figure 4B**) and LASSO variable trajectory (**Figure 4C**) plots. The LASSO regression model included 9 LASSO regression model genes: *IL-36A, AP1S3, IL1RAP, CARD14, IL1A, TNXB, CD276, SLC44A4 and IRAK1*. The risk factors derived from the LASSO risk score were plotted utilizing the package ggplot2 in R (**Figure 4D**). The RiskScore was calculated as follows:

$$\begin{array}{c} \text{RiskScore }=\text{IL}36\text{A}*\left(-0.0817\right)+\text{AP}1\text{S}3*\left(-0.0877\right)+\text{IL}1\text{RAP}*0.3167+\text{CARD}14*0.1534\;\\ +\text{IL}1\text{A}*0.1208+\text{TNXB}*0.0441+\text{CD}276*0.0468+\text{SLC}44\text{A}4*\left(-0.0387\right)\\ +\text{IRAK}1*\left(-0.1612\right) \end{array}$$

The data demonstrated that the death rate was higher in the high-risk group than in the low-risk group, and *CD276, TNXB, IL1A, CARD14* and *IL1RAP* were expressed at elevated levels in the high-risk group.

Subsequently, we conducted a prognostic KM curve analysis based on the LASSO risk score combined with the OS of the STAD group in the TCGA-STAD dataset using median value grouping (**Figure 4E**). The findings revealed a statistically significant difference in OS between the high- and low-risk groups within the STAD group of TCGA-STAD dataset (*p* < 0.001). Furthermore, we plotted a time-dependent ROC curve for the STAD group in TCGA-STAD dataset (**Figure 4F**). The study revealed that the GC prognostic risk model had an effective diagnostic capability (0.5 <AUC <0.7).

### 3.4 GO and KEGG enrichment analysis

**Figure 5:**
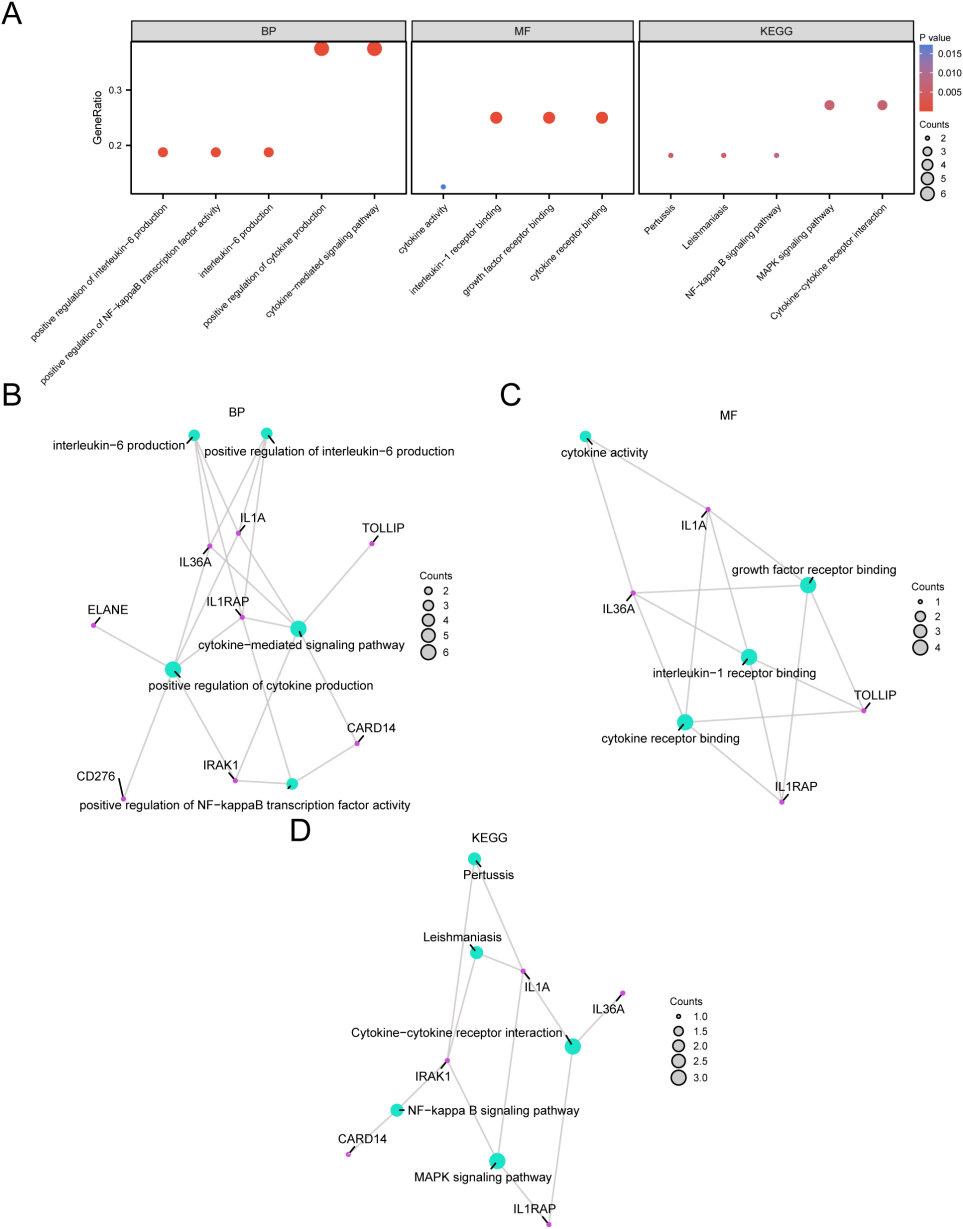
GO and KEGG Enrichment Analysis for Model genes. **(A)** The bubble chart displays the results of the GO and KEGG enrichment analyses for the model genes: BP, MF, and KEGG. **(B-D)** The network diagrams show the results of the GO and pathway (KEGG) enrichment analyses for the model genes: BP **(B)**, MF **(C)**, and KEGG **(D)**.

Through GO and KEGG enrichment analyses, we further examined the biological processes (BP), molecular functions (MF), cellular components (CC), and the relationship between biological KEGG and GC of the nine model genes. These nine model genes were used for the GO and KEGG enrichment analyses. The findings indicated that the nine model genes were primarily abundant in BP-like cytokine-mediated signaling pathways within STAD samples and in MF, such as interleukin (IL)-1 receptor binding and growth factor receptor binding. Additionally, they were enriched in biological pathways (Kyoto Encyclopedia of Genes and Genomes), including the MAPK and NF-kappa B signaling pathways. The results of the GO and KEGG enrichment analyses are illustrated in a bubble chart (**Figure 5A**). Concurrently, network diagrams of BP, MF, and biological pathways were outlined according to the results obtained from the GO and KEGG enrichment analyses (**Figures 5B–D**).

### 3.5 Gene set enrichment analysis

**Figure 6:**
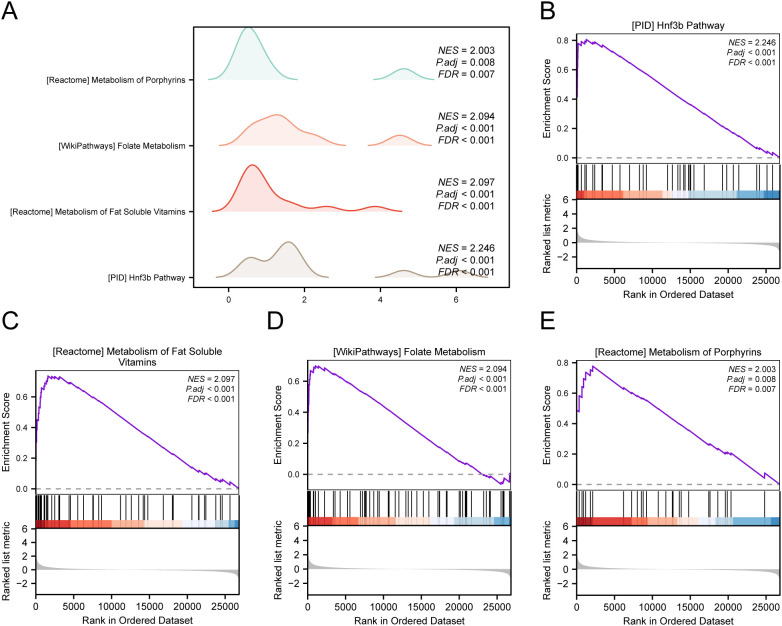
GSEA for TCGA-STAD. **(A)** Mountain map of GSEA 4 term in the TCGA-STAD dataset. **(B-E)** GSEA shows that all genes are significantly enriched in HNF3B pathway **(B)**, metabolism of fat soluble vitamins **(C)**, folate metabolism **(D)** and metabolism of porphyrins **(E)**.

To assess how the expression levels of all genes in STAD samples influenced the high and low risk of GC, GSEA was employed to examine the connections among gene expression, the biological processes in which they participate, the affected cellular components, and the molecular functions they exert, as represented through a mountain plot (**Figure 6A**). The analysis demonstrated that every gene in the TCGA-STAD dataset showed significant enrichment in biological functions and signaling pathways related to cellular metabolism, signal transduction, and regulation of gene expression (**Figures 6B–E**).

### 3.6 Gene set variant analysis

**Figure 7:**
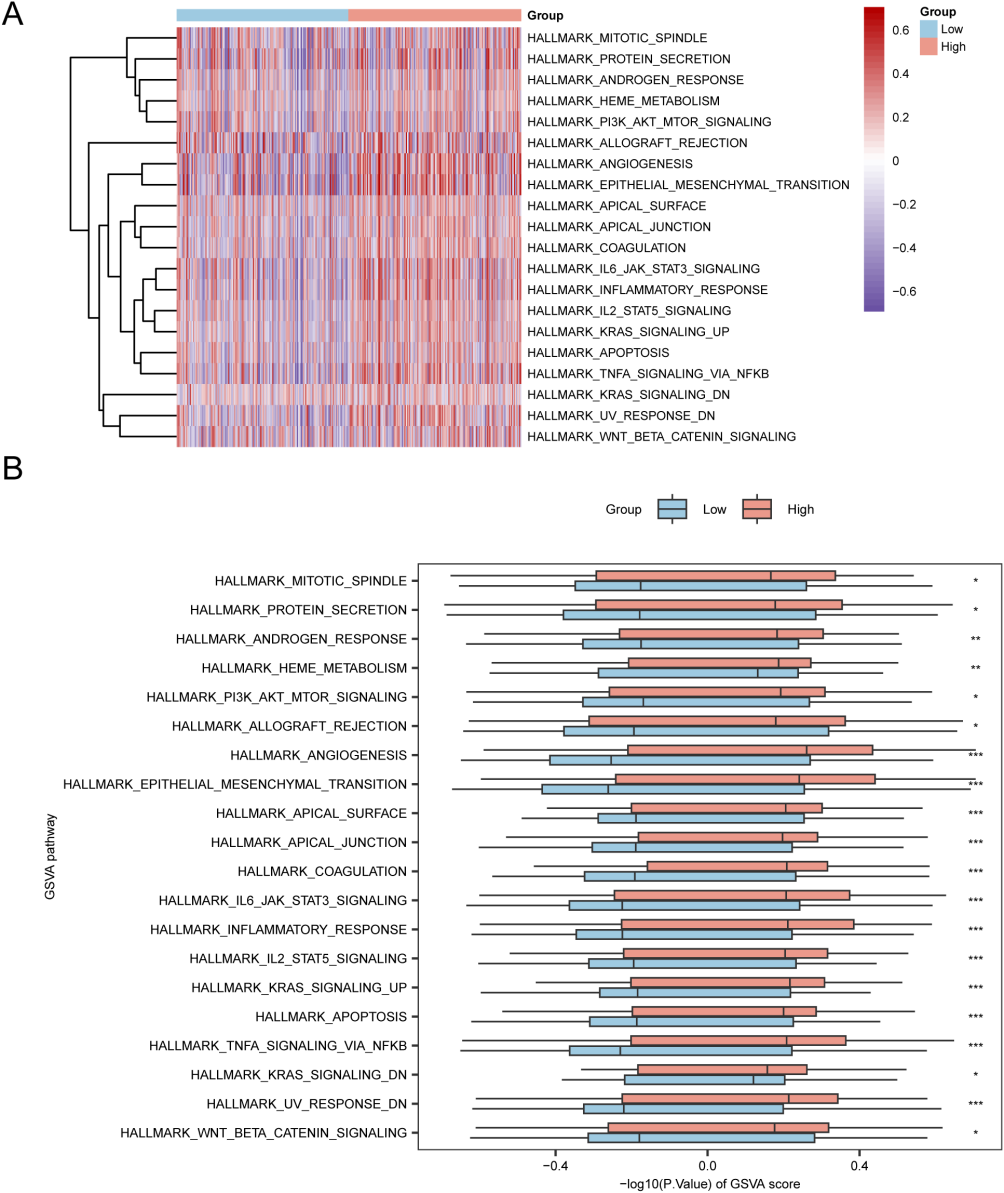
GSVA Analysis. **(A, B)** Heatmap **(A)** and group comparison of GSVA results between the high-risk and low-risk groups in the TCGA-STAD dataset **(B)**. *p value < 0.05, statistically significant; ** *p* value < 0.01, highly statistically significant; *** *p* value < 0.001, extremely statistically significant.

To investigate the differences between h.all.v7.4. symbols.gmt gene set among the high- and low-risk groups in the E dataset, we conducted GSVA for all genes in the TCGA-STAD dataset. Subsequently, we selected the top 10 positively and negatively enriched pathways with *p* values < 0.05 and log2FC rankings and analyzed the differential expression of the 20 pathways among the high- and low-risk groups, visualizing the results via a heatmap (**Figure 7A**). We then performed differential validation based on the Mann–Whitney U test and created a grouped comparison chart to display the results (**Figure 7B**).

### 3.7 Prognostic analysis of the prognostic risk model for GC

**Figure 8:**
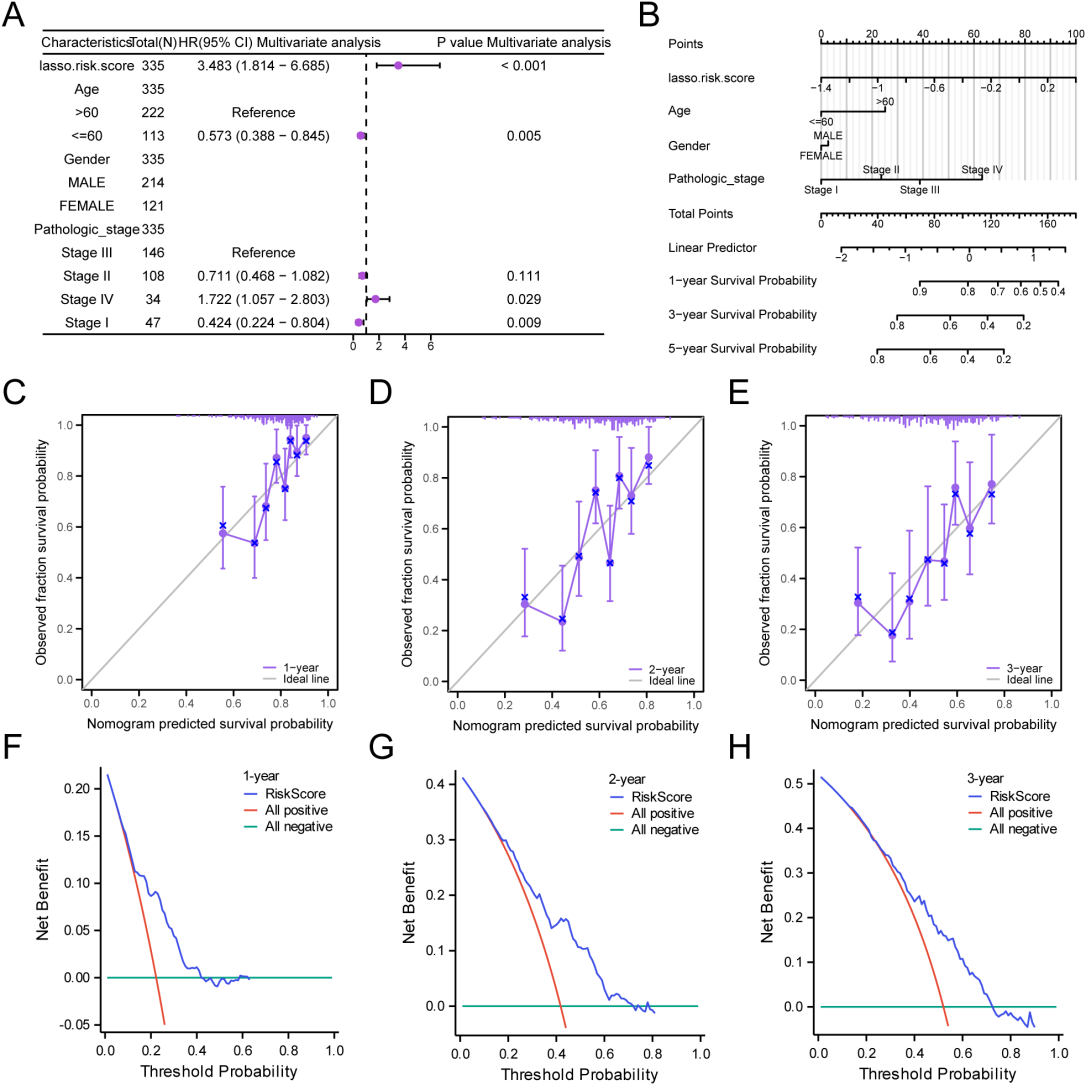
Prognostic Analysis. **(A, B)** Forest plot **(A)** and Nomogram **(B)** of the LASSO risk score and clinical information in the multivariate Cox regression model. **(C-E)** Calibration Curve for the GC prognostic risk model at 1 year **(C)**, 2 years **(D)**, and 3 years **(E)**. **(F-H)** DCA plots for the GC prognostic risk model at 1 year **(F)**, 2 years **(G)**, and 3 years **(H)**.

Based on the findings of the LASSO regression analysis, LASSO regression was performed to explore the correlation between the LASSO risk score and clinical prognosis. The results of the multivariate Cox regression analysis were visualized using a forest plot (**Figure 8A**). To further support the significance of the GC prognostic risk model, a nomogram was built using the LASSO risk score and clinical details to display the interconnections among the genes (**Figure 8B**). The results revealed that the LASSO risk score was significantly more effective in the GC prognostic risk model than other factors. Moreover, we conducted a prognostic calibration assessment for the GC risk model across 1, 2, and 3 years and plotted the calibration curves (**Figures 8C–E**). The outcomes showed that the prognostic risk model for GC had the best clinical predictive performance at the 3-year follow-up. Finally, we evaluated the clinical utility of GC prognostic risk model utilizing DCA for 1, 2, and 3 years (**Figures 8F–H**). The findings indicated that the ranking of the clinical predictive performance of our established multivariate Cox regression model was as follows: 3 years > 2 years > 1 year.

### 3.8 PPI interaction network and functional similarity analysis

**Figure 9:**
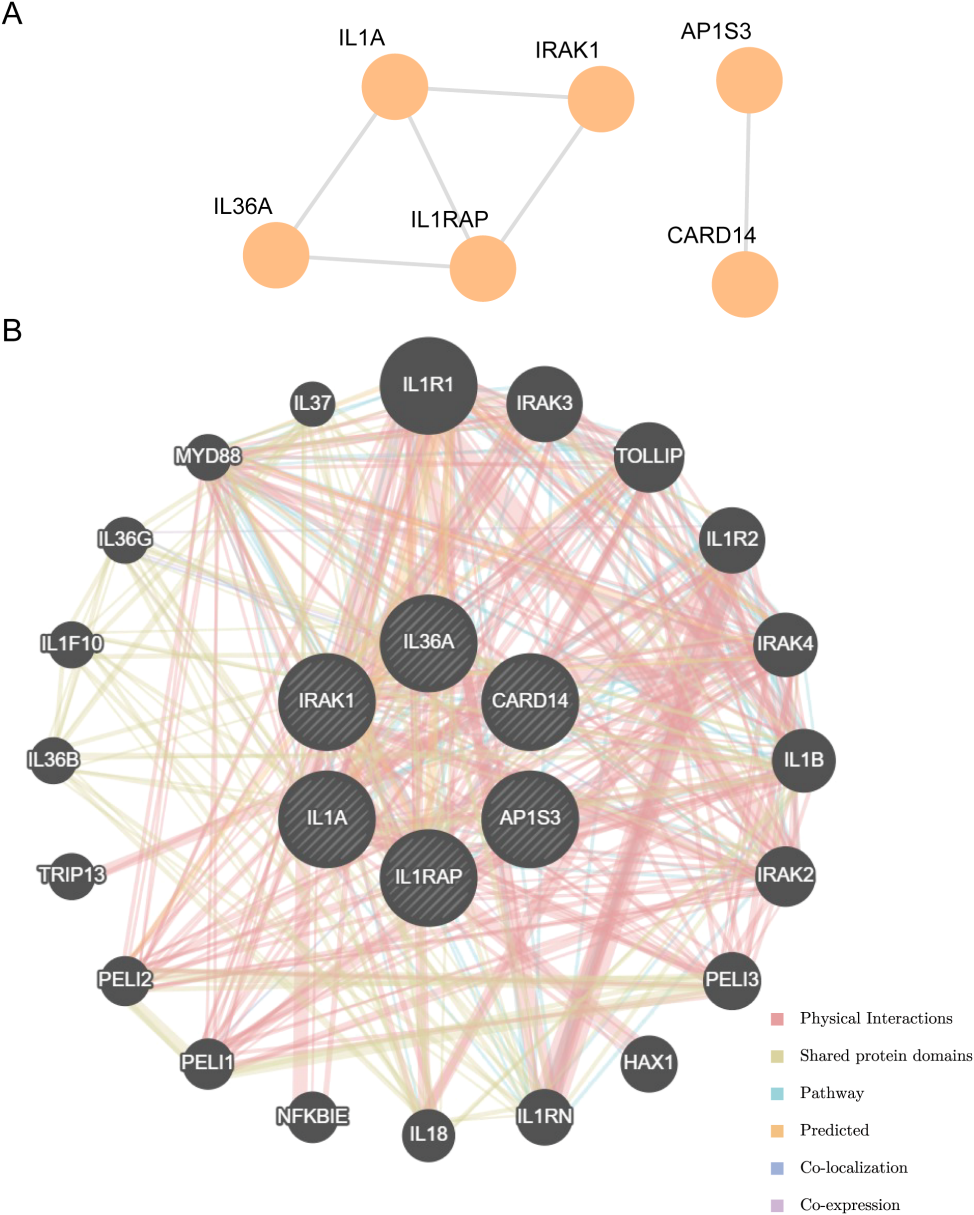
PPI interactions network and functional similarity analysis. **(A)** PPI network diagram. **(B)** The GeneMANIA website predicts the interaction network of functionally similar genes for the Hub genes.

Using the STRING database to analyze the PPI interaction network of the nine model genes, we retained only the genes that were connected to other nodes and designated them as hub genes for subsequent analysis. This resulted in the construction of a PPI network consisting of six hub genes (*IL-36A, AP1S3, IL1RAP, CARD14, IL1A*, and *IRAK1*), which were visualized using the Cytoscape software (**Figure 9A**).

The correlation of the six hub genes with other genes was analyzed using the GeneMANIA database (**Figure 9B**). The findings indicated that the six hub genes primarily exhibited co-expression, predicted interactions, physical interactions, pathways, shared protein domains, and co-localization with other genes.

### 3.9 Differential expression validation of hub genes and ROC curve analysis

**Figure 10:**
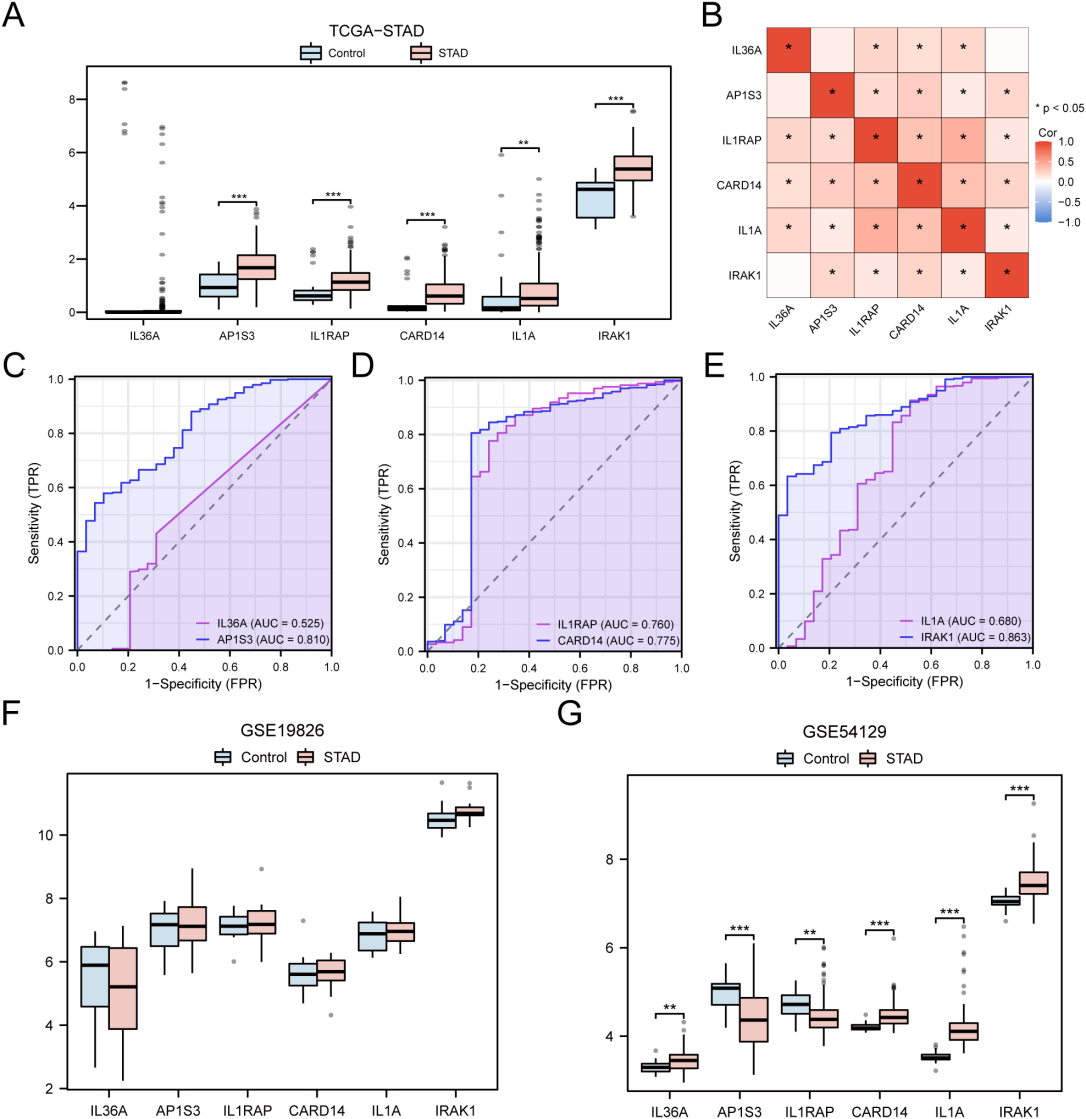
Differential Expression Validation and ROC Curve Analysis of Hub genes. **(A)** Grouped comparison chart of hub genes between STAD and Control groups in the TCGA-STAD dataset. **(B)** Correlation heatmap of hub genes in the TCGA-STAD dataset. **(C-E)** ROC curves for hub genes IL-36A and AP1S3 **(C)**, IL1RAP and CARD14 **(D)**, IL1A and IRAK1 **(E)** in the TCGA-STAD dataset. **(F, G)** Grouped comparison chart of hub genes between STAD and Control groups in the GSE19826 **(F)** and GSE54129 **(G)** datasets. * *p* value < 0.05; ** *p* value < 0.01; *** *p* value < 0.001.

The differences in the expression of the six hub genes among the STAD and control groups in the TCGA-STAD dataset were analyzed and presented in a grouped comparison chart (**Figure 10A**). Analysis of differences demonstrated that the expression of the six hub genes were statistically significant (*p* < 0.001). Correlation analysis was conducted on the expression of the six hub genes in the TCGA-STAD dataset, and a correlation heatmap was generated (**Figure 10B**). Among these, *IL1RAP* and *IL1A* showed the greatest positive association (*r* = 0.43, *p* < 0.05). Finally, using the R package pROC, ROC curves were created from the expression of hub genes in the TCGA-STAD dataset (**Figures 10C–E**). The ROC curves indicated that the four hub genes (*AP1S3, IL1RAP, CARD14*, and *IRAK1*) showed a notable level of accuracy in distinguishing the STAD group from the control group based on their expression (0.7 < AUC < 0.9).

The validation results are presented through group comparison graphs, demonstrating the expression difference analysis results of six hub genes in the STAD and control groups of the GSE19826 and GSE54129 (**Figures 10F, G**). The differential results indicated that the representation differences of the six hub genes in the dataset GSE54129 were significant and statistically meaningful.

### 3.10 Immunohistochemical results

**Figure 11:**
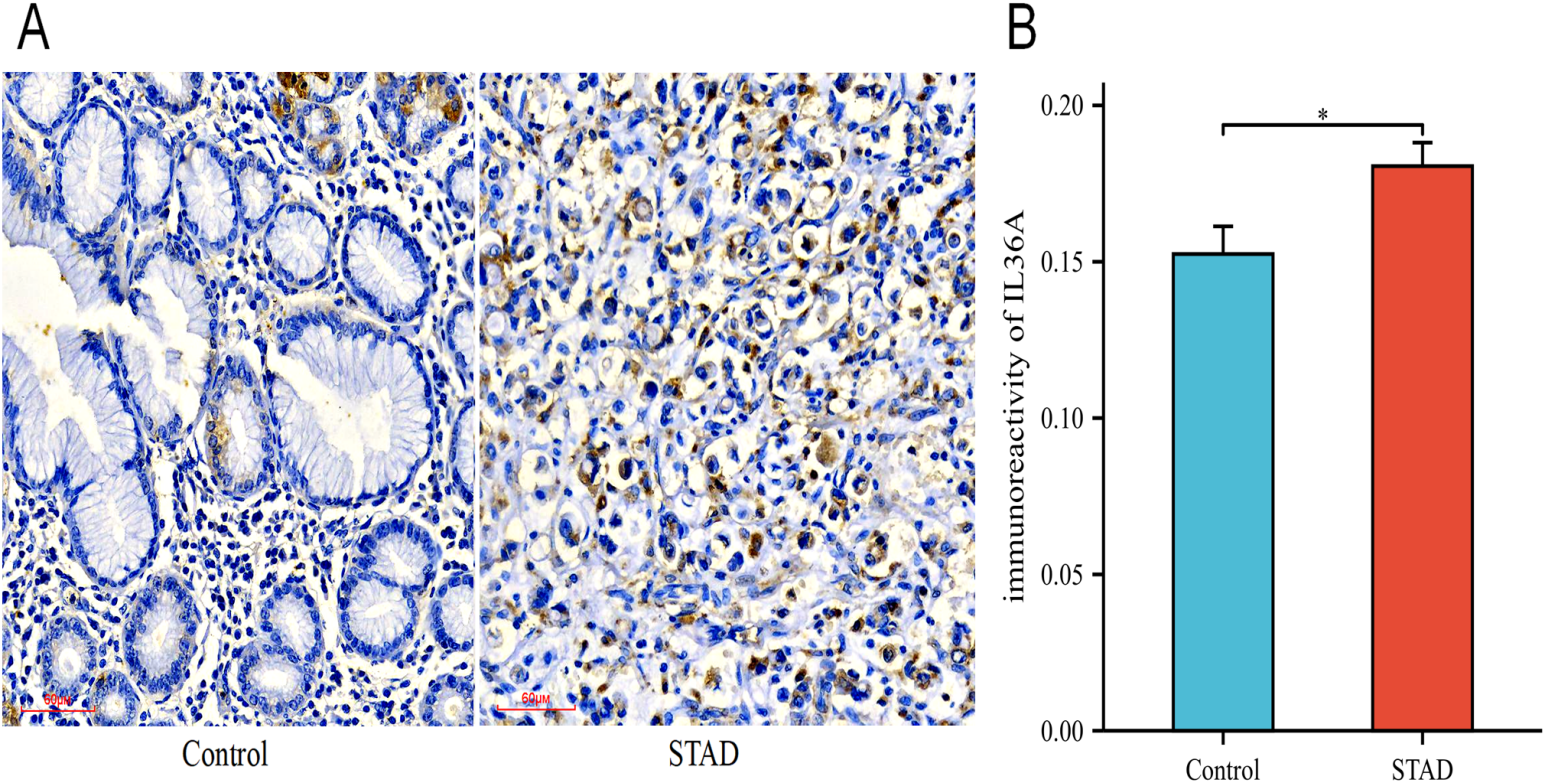
The expression levels of IL-36A protein. **(A)** The expression level of IL-36A in the Control and STAD group tissues was detected using the IHC staining method (×200). **(B)** Quantitative analysis of the average optical density (IOD/Area) of IL-36A IHC images in the Control and STAD groups was calculated using Image-J software, and the data are presented as mean ± standard deviation. * *p* < 0.05.

Among the six hub genes, previous studies have been conducted on the roles of the other five hub genes in GC (43–51), except for IL-36A. Hence, IL-36A was selected for immunohistochemistry (IHC) experiments to detect its expression in GC. In this study, a sum of 46 STAD samples and 22 adjacent normal tissue samples (controls) were collected. The specimens were embedded with paraffin and sectioned into 0.5-μm thick pieces. The expression of IL-36A protein was detected (**Figure 11A**), with positive expression indicated by brownish-yellow or brown cytoplasmic granules. The positive expression rate was assessed using the ImageJ software.

The IHC results showed that IL-36A was primarily expressed in gastric foveolar glands and stroma in the control group tissues (0.152 ± 0.04), while in the STAD tissues, IL-36A was mainly expressed in the cytoplasm (0.181 ± 0.05). For IL-36A, a statistically significant difference was found in comparison with the control group (p = 0.0186, p < 0.05) (**Figure 11B**).

### 3.11 Immune infiltration analysis

**Figure 12:**
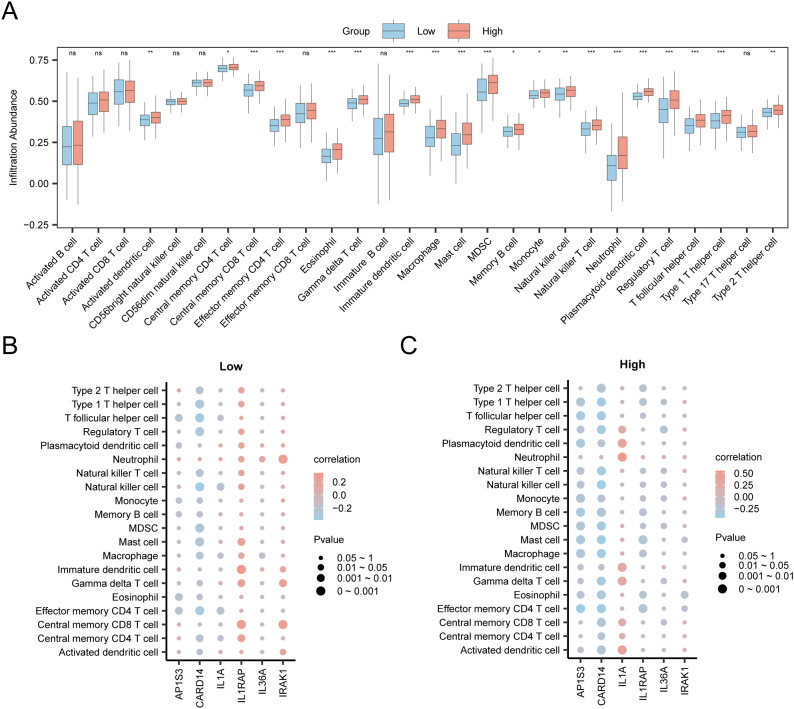
Risk Group Immune Infiltration Analysis by ssGSEA Algorithm. **(A)** Comparison of immune cells in the TCGA-STAD dataset between the high-risk and low-risk group. **(B, C)** Correlation bubble plots of immune cell infiltration abundance and hub genes in the high-risk **(B)** and low-risk **(C)**. ns *p* value ≥ 0.05, non statistically significant; * *p* value < 0.05; ** *p* value < 0.01; *** *p* value < 0.001.

In the expression matrix of the STAD group in the TCGA-STAD dataset, the ssGSEA algorithm was employed to assess the immune infiltration abundance of 28 types of immune cells within the high- and low-risk groups. First, a grouped comparison chart (**Figure 12A**) was presented to display the discrepancies in immune cell infiltration abundance across various groups. The grouped comparison chart indicated that the 20 categories of immune cells showed statistically significant differences (*p* < 0.05).

Finally, the correlation bubble plot showed the relationship between hub genes and levels of immune cells (**Figures 12B, C**). The results of the correlation bubble plot indicated that certain hub genes showed strong positive associations with specific immune cell types in the high- and low-risk groups. In the low-risk group, IL1RAP shows the strongest positive relationship with immature dendritic cells (*r* = 0.337, *p* < 0.05), whereas in the high-risk group, IL1A shows the highest positive association with neutrophils (*r* = 0.551, *p* < 0.05).

## 4 Discussion

GC has a complex etiology, is difficult to detect in its early stages, has a poor prognosis after treatment, and remains a malignant tumor with a high mortality rate, imposing a heavy economic and psychological burden on patients and their families. Patients with GC generally have a poor prognosis and low 5-year survival rate (1, 52). Important advancements have been made in recent years regarding prognostic biomarkers and molecular targets for GC. For example, prognostic risk models have been established using apoptosis-related molecules like p53, BCL-2, and Caspases-3, along with seven identified Anoikis-Related Long Non-Coding RNAs (ar-lncRNAs) (53–56). Additionally, such as the immune checkpoint PD-L1 (57), and some newer biomarkers like Human Epidermal Growth Factor Receptor 2 (HER2) (58, 59) and Fibroblast Growth Factor Receptor 2 (FGFR2) (60) have also emerged. And others existing prognostic models for GC that involve iron death-related genes (61), copper death-related genes (62), and mitochondrial-related gene models (63), these biomarkers not only serve as prognostic predictors but also provide new avenues for molecular targeted therapy. However, the efficacy and cytotoxic effects of these biomarkers in relation to targeted drugs remain unclear, there is a necessary to explore other prognostic model for GC. Our study presents a novel prognostic model associated with IL36-RGs, which has not been reported before. The IL-1 family is known to have connections with inflammation, immunity, and cancer (64); however, there has been no previous report documenting a GC prognostic model specifically focused on IL-36. As previously mentioned, some studies have confirmed a connection between the IL-36 family and the occurrence and development of tumors. However, research has typically focused on the regulatory mechanisms or predictive models of a specific factor corresponding to a single pathway. Disease occurrence and development result from an interplay between multiple factors. Therefore, this study constructed a prognostic model using IL-36RDEGs through various bioinformatics analysis methods. Firstly, the study identified 16 IL-36RDEGs. We utilized univariate Cox regression and LASSO regression analyses to identify nine feature genes, including *IL-36A*, *AP1S3*, *IL1RAP*, *CARD14*, *IL1A*, and *IRAK1* to build a prognostic risk model for GC, in which patients with high-risk scores had a significantly reduced survival duration. Calibration analysis and DCA showed that the accuracy of this prognostic risk model was sufficiently good, especially in clinical predictions over 3 years, indicating that over time, the model’s predictive ability in clinical applications gradually improved.

Regarding these nine model genes, we employed various enrichment analyses to reveal their participation in important biological processes and signaling pathways. GO and KEGG functional enrichment analyses indicated that the nine model genes primarily participate in BP that promote cytokine synthesis and cytokine-mediated signaling pathways in GC. Cytokines activate a series of intracellular signaling pathways by binding to their receptors, thereby triggering changes in the cell phenotype, metabolism, and function. This study showed that the main MF of the model genes was binding to interleukin-1 receptors and growth factor receptors, and their enrichment was predominantly observed in the cytokine-cytokine receptor interaction and the MAPK signaling pathway (KEGG). The MAPK signaling pathway can regulate interactions between cells and the microenvironment, promoting the generation of new blood vessels in tumors and interactions between tumor and immune cells (65). These observations suggest that the model genes potentially influence the tumor microenvironment of GC. Studies have shown that IL-36A is an important predictor of unfavorable prognosis in patients with non-small cell lung cancer (66). IL-36A is expressed in all types of immune and non-immune cells including T cells, neutrophils, and epithelial cells. Evidence reveals that IL-36A plays a significant pro-inflammatory biological role in the communication between different cells, such as dendritic cells, neutrophils, and epithelial cells, in the course of initiating, sustaining, and amplifying inflammation. IL-36A can also activate MAPKs and the NF-κB pathway, as reflected in the GO and KEGG analyses of the model genes in this analysis. The function of IL-36A in the tumor microenvironment is also receiving increasing attention, with one of the main mechanisms being the enhancement of immune cell infiltration through upregulation of the expression of various chemokines (67). The connection between hub genes and immune cell infiltration abundance in this study showed that IL36A positively correlated with neutrophils in the low-risk group and negatively correlated with regulatory T cells in the high-risk group. Earlier research has indicated that IL-36A could be closely linked to the tumor immune microenvironment (68). Thus, IL-36A is a potentially effective target for clinical immunotherapy of GC. Our study revealed that IL-36A, as a factor in the GC prognostic risk model, is being reported for the first time. The validation of the differential analysis results demonstrated statistical significance for the Hub genes in the GSE54129 dataset. However, the differential analysis of IL-36A in the TCGA-STAD dataset showed no statistical significance, and there is limited research on its expression in GC tissues. Given the existing research background, it is crucial to further clarify its expression in GC tissues. This study detected the expression of IL-36A in GC using immunohistochemistry. The analysis demonstrated a statistically significant difference in the expression between the STAD and Control groups. In summary, this study provides a theoretical foundation for studying the mechanism of immune action of IL-36A in GC.

Research in GC indicated that AP1S3 plays a role in enhancing the development of GC (43, 44). IL1RAP shows higher expression in GC tissues than in non-cancerous tissues and is involved in the occurrence of GC and regulation of the inflammatory process (45). The findings on AP1S3 and IL1RAP were consistent with the analysis of differentially expressed hub genes in this study, and the ROC curve analysis showed good accuracy. In the diffuse GC group, elevated CARD14 expression was significantly associated with worse patient prognosis (46). Malignant cells, tumor-infiltrating immune cells, and stromal cells can express IL1A (47, 69), which is overexpressed in GC and is closely related to the clinical features of patients with GC (48, 49, 70). IRAK1 is associated with negative prognosis and invasiveness of GC (50, 51). Research findings on CARD14, IL1A, and IRAK1 suggest that they can serve as biomarkers for GC prognosis, which concurs with the outcomes of this investigation. Western blotting and immunohistochemical experiments have indicated that TNXB is overexpressed in patients with gastric adenocarcinoma with lymph node metastasis (71), which is linked to a lower survival rate in these patients and can serve as a prognostic marker. CD276 and SLC44A4 have also been confirmed as prospective targets for immune checkpoints, diagnosis, prognosis, and treatment of tumors (72–74). In summary, the study reveals that all nine model genes were associated with tumors or inflammation, highlighting their potential functions and prognostic abilities in GC. This provides strong support for the reliability of this study and is consistent with the outcomes of the prognostic risk model.

To better elucidate the interactions among model genes, we created a PPI network and identified six hub genes. Hub genes exhibit physical interactions, pathways, and co-expression. By analyzing the relationship between hub genes and differential immune cell infiltration abundance, it was observed that in the low-risk group, IL1RAP exhibited a positive relationship with the levels of immature dendritic cell infiltration. The innate immune system, especially immature dendritic cells, can be activated by recognizing DAMPs released by tumor cells through pattern recognition receptors, triggering metabolic changes in dendritic cells and playing a role in cancer immune surveillance (75). IL1A levels were positively correlated with the abundance of neutrophil infiltration in high-risk samples. Neutrophils are a major component of the tumor microenvironment. Studies have shown that neutrophils interact with GC cells, promoting their invasion and migration of GC cells through the induction of the epithelial-mesenchymal transition (EMT) and activation of the ERK pathway (76). Neutrophils may also support the development and progression of GC by facilitating angiogenesis and reducing antitumor T cell activity (77, 78), suggesting that neutrophils contribute to the occurrence and development of GC. Hub genes may influence the tumor microenvironment or patient prognosis by affecting the abundance or activity of specific immune cells. These findings provide important clues for personalization of immunotherapy.

GSEA showed that all genes within the STAD group were primarily focused on biological processes and signaling pathways related to cell development and metabolism. Changes in cell development and metabolism, which can trigger alterations in the tumor microenvironment, have gained increasing attention (79–83). These pathways may be directly connected to and closely related to the occurrence, development, and changes in the GC microenvironment, thus providing important clues for mechanistic research and potential therapeutic strategies for GC. The outcomes of the enrichment analysis of these pathways through GSVA can reveal biological processes or signaling pathways that exhibit notable variations between the high- and low-risk groups, such as angiogenesis, EMT, and inflammatory response pathways, which could be linked to the onset and progression of GC. These findings provide guidance for future research on these mechanisms.

## 5 Conclusion

The prognostic risk model constructed using IL-36RDEGs is an independent prognostic risk factor for GC and can be used to assess the prognosis of patients with GC. This model provides a powerful tool for clinical practice, as it can identify high-risk patients and optimize treatment strategies, thereby leading to a better overall prognosis for patients with GC. The expression of critical genes was initially validated through immunohistochemical staining. However, some limitations remain unresolved. For instance, the sample size was relatively small, and there is a deficiency of extensive, multicenter clinical samples for further external validation. Although we validated the expression of core genes and their ability to distinguish GC from normal tissues using two independent datasets, GSE19826 and GSE54129 from the GEO databases, the public GEO datasets generally lack comprehensive clinical prognostic follow-up data. Consequently, a systematic assessment of the overall prognostic predictive ability of the model using independent external cohorts is not yet possible. Therefore, the generalizability and clinical applicability of the model require further validation in independent cohorts with complete prognostic information. Additionally, some potential influencing factors may have been inadequately considered due to limitations in data sources and the analytical methods employed. The model achieved an AUC value of just 0.584 for the 3-year overall survival rate, suggesting a constrained predictive capability. It is necessary to optimize and enhance the prognostic predictive efficacy of the model in the future by increasing sample sizes and refining multi-center clinical cohort data. We intend to gather and analyze independent cohorts with clinical prognostic information to improve the model’s utility and generalizability. Overall, this study provides new evidence for the prognostic direction of GC, which can act as a potential therapeutic target for further mechanistic validation and clinical evaluation.

## Data Availability

The datasets presented in this study can be found in online repositories. The names of the repository/repositories and accession number(s) can be found in the article/**Supplementary Material**.
