# Supplementary material for: IL-36-related genes predict prognosis of gastric cancer
Source: Front Oncol. 2025 Jun 18;15:1566993. doi: 10.3389/fonc.2025.1566993 (PMC12213462; doi:10.3389/fonc.2025.1566993)
Supplement: Supplementary file 1 [file Table1.docx]

Supplementary Material

# Supplementary Figures and Tables

**TABLE 1 IL-36 relate genes list**

| **IL-36 relate genes list** | | | |
| --- | --- | --- | --- |
| IL36A | SELE | IL1RN | FKBPL |
| IL36B | IL17A | STAMBP | PRRT1 |
| IL36G | SERPINA3 | CD276 | ENSG00000273171 |
| IL36RN | TNIP1 | USP2 | IRAK1 |
| IL1RAP | C1orf141 | OTUB1 | TRAF6 |
| IL1RL2 | LCE3C | RNF125 | TOLLIP |
| IL1A | LCE3B | AGER | CAMP |
| AP1S3 | IL1F10 | VTN | S100A7 |
| ELANE | NEU1 | SARM1 | NFKB1 |
| CARD14 | TNXB | BAG6 | PTX3 |
| TNF | MYD88 | SLC44A4 | PGLYRP2 |

**TABLE 2 IL-36DEGs**

| **IL-36DEGs** | | | |
| --- | --- | --- | --- |
| IL36A | CARD14 | USP2 | PRRT1 |
| AP1S3 | IL1A | SARM1 | IRAK1 |
| IL1RAP | TNXB | BAG6 | TOLLIP |
| ELANE | CD276 | SLC44A4 | PTX3 |
